# Biological toxicity of sulfamethoxazole in aquatic ecosystem on adult zebrafish (*Danio rerio*)

**DOI:** 10.1038/s41598-024-59971-y

**Published:** 2024-04-24

**Authors:** Jie Zhou, Xiao Yun, Jiting Wang, Qi Li, Yanli Wang, Wenjing Zhang, Zhicheng Fan

**Affiliations:** Lab of Aquatic Animal Nutrition & Environmental Health, Shandong Agricultural University, 61 Dazing Street, Tai’an, 271018 Shandong China

**Keywords:** Sulfamethoxazole, Zebrafish, Oxidative stress, Histopathology, Intestinal microbiota, Ecology, Immunology, Environmental sciences

## Abstract

This study evaluated the impacts of sulfamethoxazole (SMX) on antioxidant, immune, histopathological dynamic changes, and gut microbiota of zebrafish. SMX was carried out five groups: 0 (C), 3 mg/L (T3), 6 mg/L (T6), 12 mg/L (T12), and 24 mg/L (T24), with 5 replicates per group for an 8-weeks chronic toxicity test. It was found that SMX is considered to have low toxicity to adult zebrafish. SMX with the concentration not higher than 24 mg/L has no obvious inhibitory effect on the growth of fish. Under different concentrations of SMX stress, oxidative damage and immune system disorder were caused to the liver and gill, with the 12 and 24 mg/L concentration being the most significant. At the same time, it also causes varying degrees of pathological changes in both intestinal and liver tissues. As the concentration of SMX increases, the composition and abundance of the gut microbiota in zebrafish significantly decrease.

## Introduction

Antibiotics are widely used to treat and prevent human diseases, and can also promote animal growth and improve animal health in aquaculture and animal husbandry^1^. However, the widespread use of antibiotics causes serious pollution to the aquatic environment, and the excessive use of antibiotics poses a huge threat to aquatic ecosystems and organisms^2^. According to reports, quinolones, sulfonamides, tetracyclines, and macrolides have genetic toxicity to fish, and they accumulate biologically in water such as rivers and surface water, as well as in fish tissues^3^. Even if the concentration of antibiotics is very low, antibiotic resistant strains will be produced, producing non-target toxicity to aquatic body. However, there are currently less reports on the impact on fish than on microorganisms, and further research is needed.

Sulfonamide has become one of the most widely used antibiotics due to its broad-spectrum and high efficacy^4^. Sulfamethoxazole (SMX) is a type of sulfonamide compound widely present in water such as drinking water, wastewater, and surface water. Due to the difficulty in hydrolysis and biodegradation and its N4 acetylated metabolites, it can accumulate in aquatic ecosystems^5,6^. Sulfonamides residues exist in the waters of the Pearl River and Yangtze River Delta in China, with a concentration of 500 µg/L^7^. Like other antibiotics, the excessive use and contamination of sulfonamide can have many adverse consequences for humans and animals. Research has shown that long-term exposure to sulfonamide can lead to urinary and hematopoietic dysfunction in humans^8^. At the same time, it can also have multiple negative effects on aquatic organisms, inhibiting the metabolism of chloroplasts, such as protein synthesis and photosynthesis, affecting cell growth^9,10^, and affecting fish behavior and reproduction, resulting in a decrease in spontaneous swimming activity and an increase in heart rate of zebrafish^11^. Even low concentrations of SMX (1 µg/L) have significant toxic effects on zebrafish embryos, leading to embryonic malformations and shortened body length^12^.

Although the residual antibiotic content is not high, most antibiotics have a biological amplification effect. Even small doses of antibiotics remaining in aquatic environments will accumulate layer by layer in the food chain, reaching the highest level of the food chain and posing a threat to human health. Therefore, this study focuses on the most commonly detected antibiotic sulfonamides in surface water as the target antibiotic, exposing zebrafish to different doses of sulfonamides in water to evaluate their toxicity. The research results will provide a threshold concentration for the aquatic ecological risk of the target antibiotic, providing a scientific basis for the development of antibiotic aquatic ecological risk assessment.

## Material and methods

### Testing animals

The fish used in this experiment is wild type zebrafish of AB strain, purchased from the zebrafish breeding base of Shandong Agricultural University, with a body length of 33.57–35.42 mm and the average body weight was 0.312 ± 0.029 g. The study was approved by Ethics Committee of Shandong Agricultural University, all methods were carried out in accordance with relevant guidelines and regulations (Approval number: SDAUA-2020-025). This study was carried out in compliance with the ARRIVE guidelines.

### Test design

The chronic toxicity test of zebrafish was conducted in accordance with GB-T21806-2008. Building upon the results of previous acute toxicity tests, this test was carried out with 5 SMX groups: 0 mg/L (C), 3 mg/L (T3), 6 mg/L (T6), 12 mg/L (T12), and 24 mg/L (T24). Each treatment consisted of five replicates, with each replicate containing twenty zebrafish.

The domestication and breeding methods of zebrafish were based on the methods of Yun Xiao et al.^13^. In the feeding experiment, to prevent the impact of other pollutants on this experiment, it is necessary to clean up excrement in a timely manner and replace the exposed liquid every 48 h to ensure a constant concentration of SMX in the water. The number of dead individuals was recorded.

### Sample collection

After the chronic toxicity test, zebrafish were subjected to a 48-h fasting period, then anesthetized with MS-222 and dissected under a microscope to collect samples. The zebrafish were placed on an ice plate during dissection, and liver, gill, and intestine tissues were carefully extracted. These tissues were then washed with pre-cooled double distilled water, dried using filter paper, and subsequently transferred into separate centrifuge tubes of 2.5 mL and 5 mL capacities respectively. All samples were stored in a refrigerator at -80 ℃ for subsequent testing. Specifically, liver, gill, muscle, and intestine tissues were collected.

### Index determination

#### Growth index

After the feeding experiment, weighed and recorded the weight and quantity of zebrafish in each tank for the calculation of growth indicators.

Specific growth rate (SGR, %) = 100 × [ln (W_t_)–ln (W_0_)]/t; Among them, W_0_ and W_t_ are the initial weight and final weight of the fish, respectively, and the “t” is the number of days of the experiment.

#### Determination of antioxidant related enzyme activity in liver and gill

Accurately weighed each tissue and prepared a tissue homogenate for measuring enzyme activity. Added physiological saline at a weight (g): volume (mL) ratio of 1:9. Ground the homogenate in a handheld homogenizer in an ice bath to produce a 10% homogenate. Centrifuged the homogenate at 4 ℃, 2500 r/min, 15 min, and store the supernatant at − 20 ℃ for enzyme activity measurement. Determination of SOD, CAT, GSH Px activity and MDA content in liver and gill using a reagent kit.

#### qRT-PCR analysis of genes in liver and gill

Total RNA from gills and liver was isolated using TRIzol reagent (Tianjin, China). Specific primers for genes were designed based on the relevant cDNA sequences of zebrafish (Table1). The specific process and analysis method can be found in the reported of Yun et al.^13^.

**Table 1:** Information of primers used for mRNAs qRT-PCR.

Gene name	Primer name	Primer sequence (5′ to 3′)
*β-actin*	*β-actin-F*	ATGGATGAGGAAATCGCTGCC
	*β-actin-R*	*CTCCCTGATGTCTGGGTCGTC*
*Cu/Zn-sod*	*Cu/Zn-sod-F*	*GGTCCGCACTTCAACCCTCA*
	*Cu/Zn-sod-R*	*TTCCTCATTGCCACCCTTCC*
*GPX*	*GPX-F*	*AGGCACAACAGTCAGGGATT*
	*GPX-R*	*CAGGAACGCAAACAGAGGG*
*CAT*	*CAT-F*	*CAAGGTCTGGTCCCATAAA*
	*CAT-R*	*TGACTGGTAGTTGGAGGTAA*
*IL-1β*	*IL-1β-F*	*GAGTCCGTCAAATGTCCCG*
	*IL-1β-R*	*CGCTCGGTGTCTTTCCTGT*
*IL-8*	*IL-8 -F*	*GACCAGCAAAATCATTTCAGTG*
	*IL-8 -R*	*CGTGGATCTACAGCCAGACCTC*
*TNF-α*	*TNF-α-F*	*GCTGGATCTTCAAAGTCGGGTGTA*
	*TNF-α-R*	*TGTGAGTCTCAGCACACTTCCATC*

#### Preparation and observation of stained tissue sections of liver and intestine

The liver and intestinal were fixed with 4% paraformaldehyde solution, and then dehydrated step by step with ethanol. The xylene solution was transparent and paraffin embedded. After continuous sectioning, dewaxing, and haematoxylin eosin (H.E) staining, the neutral gum was used to seal the film after drying. Finally, the film was observed and photographed under the optical microscope. Measure and statistically analyze the height, width, depth, and thickness of intestinal villi in the collected images using Image J software, and use the average of the results as the measurement result for the tissue sample.

#### Analysis of gut microbiota

The entire gastrointestinal tract of zebrafish was dissected, followed by extraction of its contents for total DNA isolation, PCR amplification, and sequencing analysis of the gut microbiota. The detailed methodology and analytical approach can be found in the study conducted by Yun et al.^13^.

#### Sulfamethoxazole residue analysis

The pre-treatment method for muscle samples was based on the method of Yun et al.^13^. The analysis of SMX was conducted at Shandong Shitong Testing and Evaluation Technology Service Co., Ltd.

### Statistics and analysis

Statistical analysis was conducted on the experimental data using the Statistical Product and Service Solutions (SPSS, Version 26.0. URL link: https://www.ibm.com/spss) software, and all data were expressed as mean ± standard deviation. Single factor analysis of variance (ANOVA) and tukey's multiple comparisons were performed on the data, with P < 0.05 being the significant difference.

## Results

### Growth results

**Figure 1:**
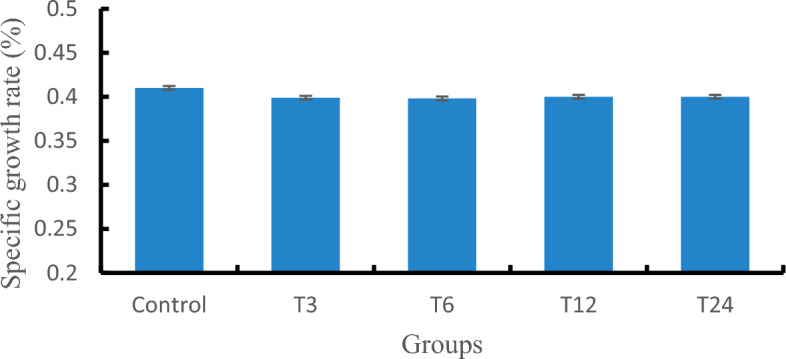
The specific growth rate of zebrafish. Different letters in the same column indicate significant differences (*P* < 0.05), and the same letters or no letters indicate insignificant differences (*P* > 0.05).

After an 8-week chronic exposure test, the specific growth rates of each group are shown in Fig. 1. The results indicate that there was no statistically significant difference (*P* > 0.05) observed between the experimental and control groups.

### Antioxidant related enzyme activity

#### Antioxidant related enzyme activity in liver tissue

**Figure 2:**
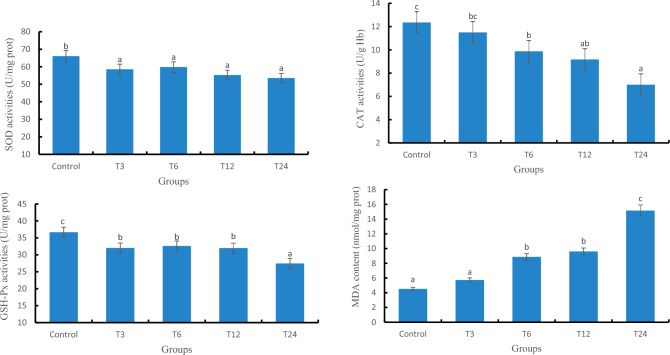
The antioxidant related enzyme activities in the liver of zebrafish. Different letters on the bars indicate significant differences (*P* < 0.05), the same letters or no letters indicate insignificant differences (*P* > 0.05), the same below.

As depicted in Fig. 2, the T24 group exhibited significantly lower activities of SOD and GSH-Px compared to the control group (*P* < 0.05), with reductions of 23.30% and 25.11%, respectively. Except for the T3 group, all other test groups demonstrated a significant decrease in CAT activity when compared to the control group *(P* < 0.05). The MDA content in the T6, T12, and T24 groups displayed significant differences from the control group (*P* < 0.05), with increases of 96.65%, 112.88%, and 236.19%, respectively.

#### Antioxidant related enzyme activity in gill tissue

**Figure 3:**
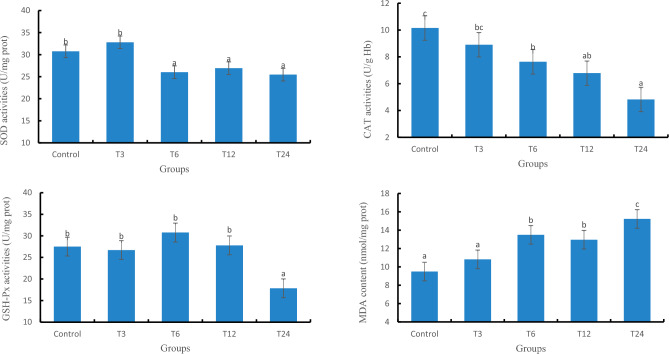
The antioxidant related enzyme activities in the gills of zebrafish.

The activities of SOD, GSH-Px, and CAT gradually decrease with the increase in SMX exposure concentration, as depicted in Fig. 3; meanwhile, MDA exhibits a progressive upward trend.

### The mRNA levels of antioxidant and immune related genes

#### The relative expression of antioxidant and immune related genes in liver

**Figure 4:**
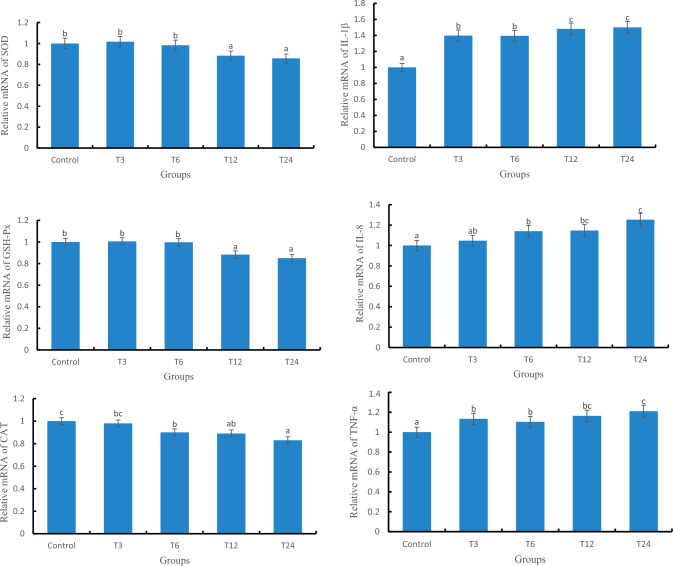
Relative expression of antioxidant and immune genes in the liver of zebrafish.

The relative expression levels of SOD, GSH-Px, and CAT genes in the T24 group were significantly lower than those in the control group (*P* < 0.05), as depicted in Fig. 4. Additionally, a significant difference (*P* < 0.05) was observed between the T3 and T24 groups. Moreover, with increasing exposure concentration of SMX, there was an upward trend in the relative expression levels of IL-1β, IL-8, and TNF-α genes; furthermore, a significant difference (*P* < 0.05) existed between the T24 group and the control group.

#### The relative expression levels of antioxidant and immune related genes in gill

**Figure 5:**
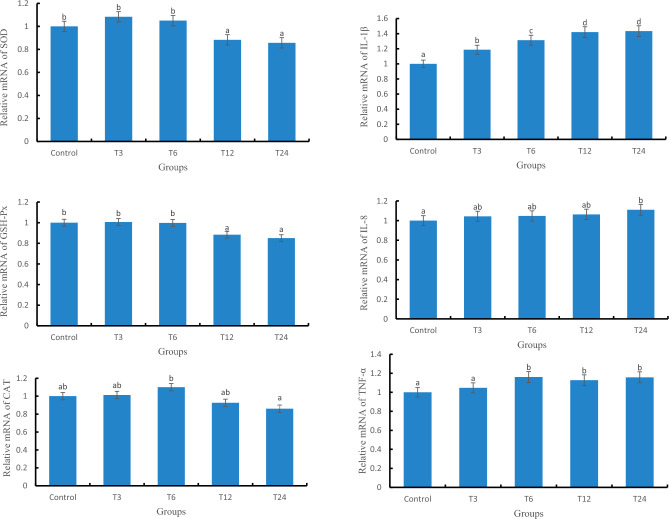
Relative expression of antioxidant and immune genes in the gills of zebrafish.

As depicted in Fig. 5, the gene expression levels of SOD, CAT, and GSH-PX exhibited a declining trend with increasing SMX exposure concentration. Moreover, a significant difference (*P* < 0.05) was observed between the T24 group and the control group. Simultaneously, as the SMX exposure concentration increased, there was an upward trend in the gene expression level of IL-1β, IL-8, and TNF-α. Additionally, a significant difference (*P* < 0.05) was found between the T24 group and the control group.

### Morphology

#### Morphology of the intestinal tissue

**Figure 6:**
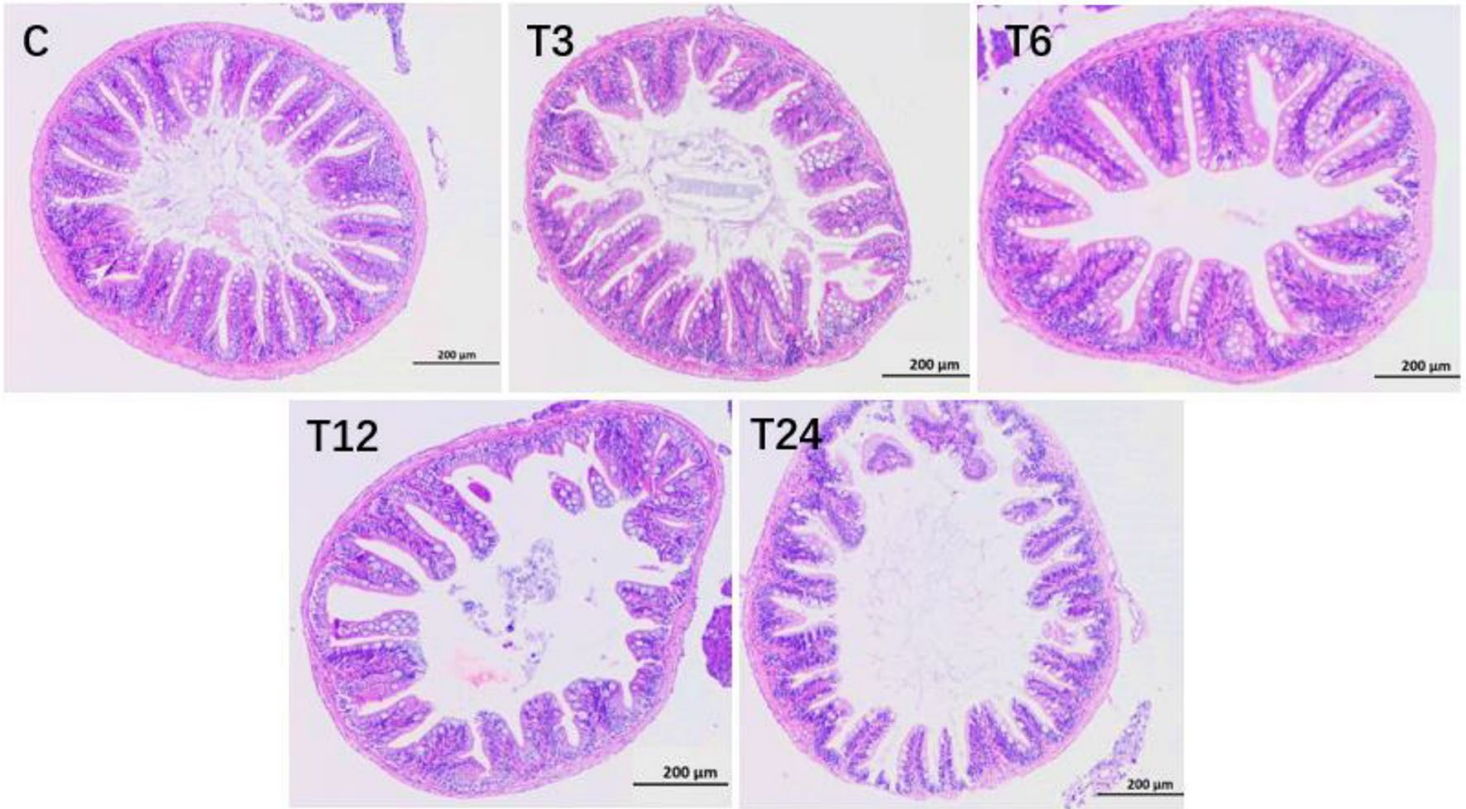
Morphology of the intestinal tissue.

The intestinal tissue section is depicted in Fig. 6. In comparison to the control group, the T6 group exhibited an increase in goblet cell count, which displayed an upward trend with increasing SMX concentration. The data for zebrafish intestinal villus height, villus width, crypt depth, and muscle layer thickness are presented in Table 2. As the SMX concentration increased, there was a decrease in villus height across all groups, particularly evident in the T24 group where it decreased significantly by 41.13% (*P* < 0.05). The T3, T6, and T12 groups demonstrated significantly higher villus width compared to the control group (P < 0.05). A significant difference was observed between the muscle layer thickness of the T24 group and that of the control group (*P* < 0.05), with a decrease of 40.65%.

**Table 2:** The intestinal morphology of zebrafish.

Items	Control (0 mg/L)	T3 (3 mg/L)	T6 (6 mg/L)	T12 (12 mg/L)	T24 (24 mg/L)
Villus height (μm)	84.62 ± 3.20^b^	104.39 ± 12.18^c^	82.82 ± 2.70^b^	64.53 ± 2.45^a^	59.96 ± 1.02^a^
Villus width (μm)	27.36 ± 0.75^a^	34.33 ± 2.77^b^	39.04 ± 1.04^b^	34.04 ± 0.84^b^	25.17 ± 1.97^a^
Crypt depth (μm)	15.82 ± 0.88	16.39 ± 1.17	14.79 ± 0.92	14.24 ± 1.24	14.67 ± 1.06
Muscular thickness (μm)	11.12 ± 0.59^b^	8.10 ± 2.34^ab^	7.06 ± 0.79^ab^	7.49 ± 1.03^ab^	6.60 ± 0.49^a^

Values with different letters are significantly different (P < 0.05). The absence of letters indicates no significant difference between treatments.

#### Morphology of the liver tissue

**Figure 7:**
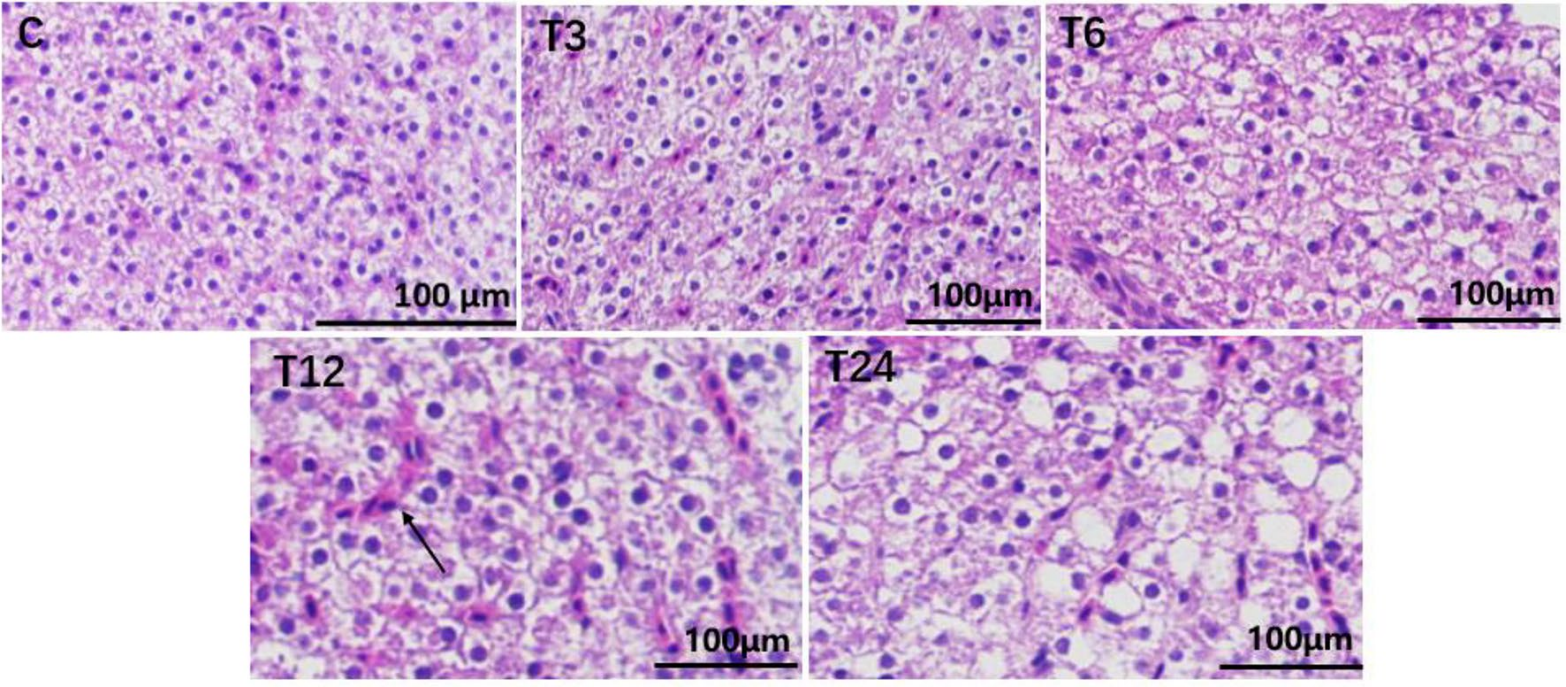
Liver morphology of zebrafish.

As shown in Fig. 7, compared with the blank group, with the increase of SMX concentration, the number of vacuolar cells increased; And there were phenomena such as nuclear deformation and nuclear pyknosis, with the T24 group being the most significant. As the concentration of SMX increased, the number of red blood cells in liver tissue increased and congestion became severe. All experimental groups showed significant differences from the control group (*P* < 0.05).

### Gut microbe

#### Sequencing depth evaluation

**Figure 8:**
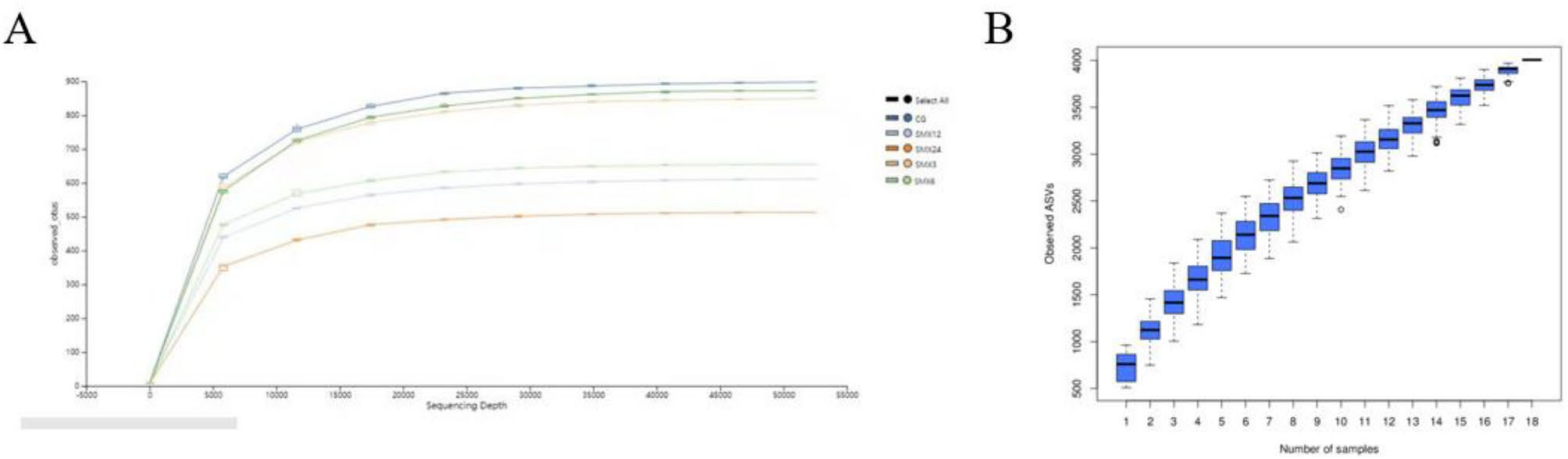
Dilution curve and Cumulative box chart of Alpha diversity of intestinal flora. CG, SMX3, SMX6, SMX12 and SMX24 refer to the control, 3, 6, 12 and 24 mg/L concentration groups, respectively.

As shown in Fig. 8, the dilution curves of each group show that as the number of samples increases, the curves tend to flatten out, indicating that the sequencing data volume is gradually reasonable, and more data volumes will not have a significant impact on the alpha diversity index. And the trend of the box chart shows an increase in sample size, indicating that a large number of species have been discovered in the community.

#### Alpha diversity analysis

**Figure 9:**
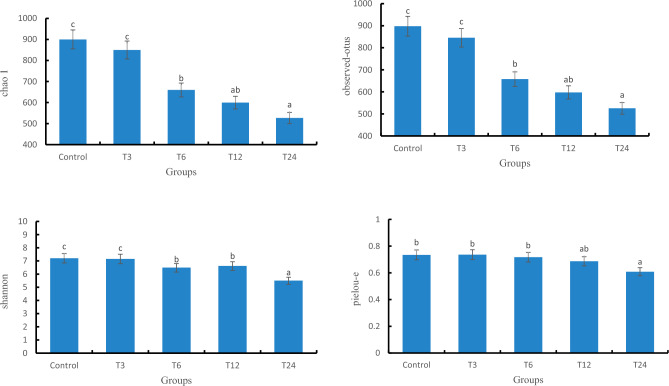
The alpha diversity index of the intestinal flora.

As shown in Table 3, the Chao1, Shannon, Observed-otus, and Pielou-e indices of the T24 group were significantly lower in numerical values than the control group (*P* < 0.05), and it can be seen from Fig. 9 that there was a downward trend among the groups, reflecting that as the SMX concentration increased, the number of low abundance species became fewer and the species uniformity became worse, and the number of visually observable species became fewer and fewer, the community diversity became lower, and the species distribution became more uneven.

**Table 3:** Significance P-value of one-way ANOVA for gut microbiota diversity index.

	T3	T6	T12	T24
Chao1 CG	0.513	0.050	0.050	0.050
Observed_otus CG	0.275	0.050	0.050	0.050
Pielou_e CG	0.827	0.127	0.513	0.050
Shannon CG	0.827	0.050	0.050	0.050

#### Venn diagram analysis of species

**Figure 10:**
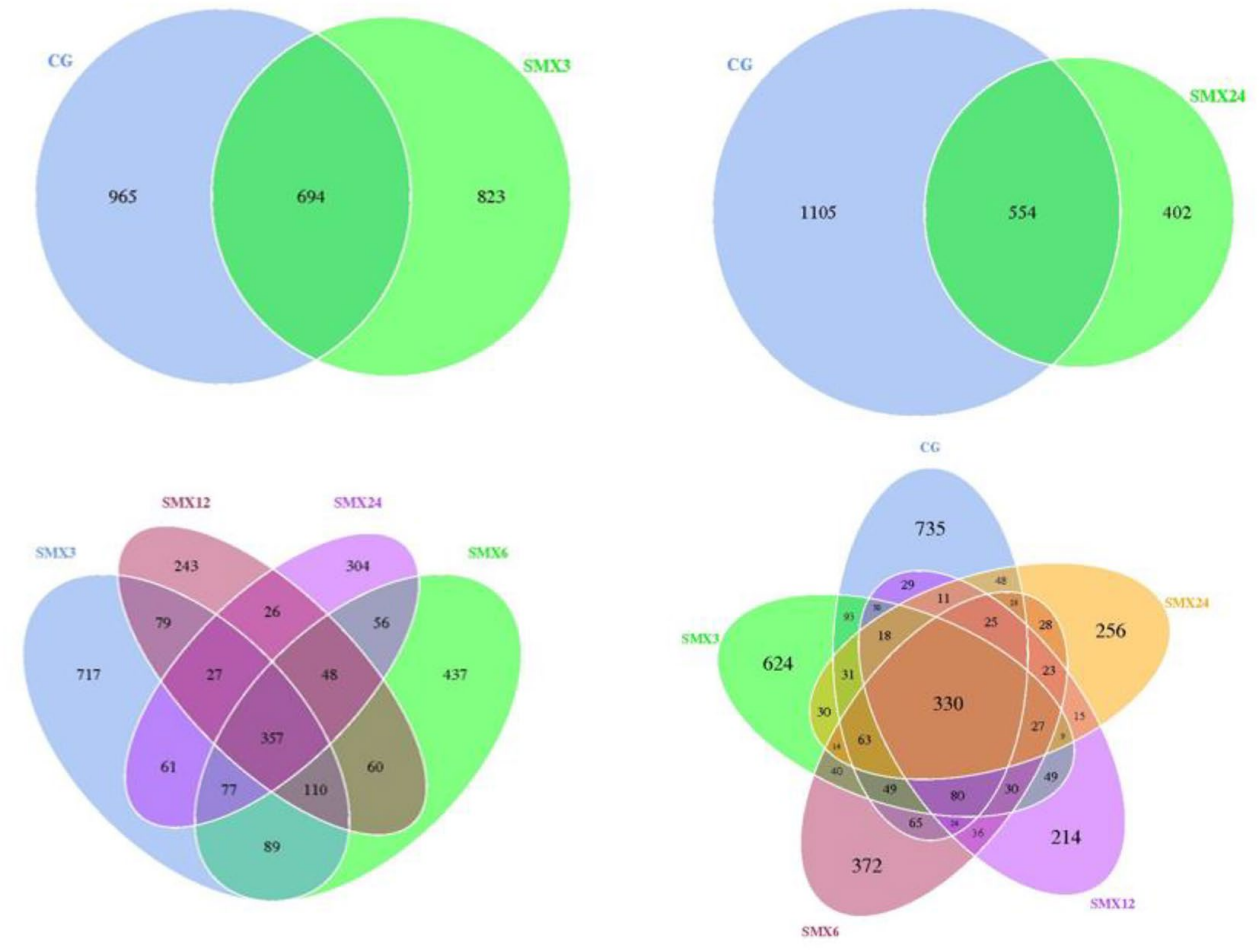
Venn diagram of different samples.

The Fig. 10 is a Venn diagram of the common and unique numbers of gut microbiota. Overall, SMX has a significant impact on the gut microbiota, with a significant decrease in bacterial richness and diversity in the test group compared to the control group (*P* < 0.05). The number of ASVs shared by each group is 330, among which the number of ASVs unique to T3, T6, T12, and T24 groups is 624, 372, 214, and 256. This result indicates that different concentrations of SMX affect the relative content of certain microbial populations.

#### Similarity and differences in the gut microbiota of zebrafish

**Figure 11:**
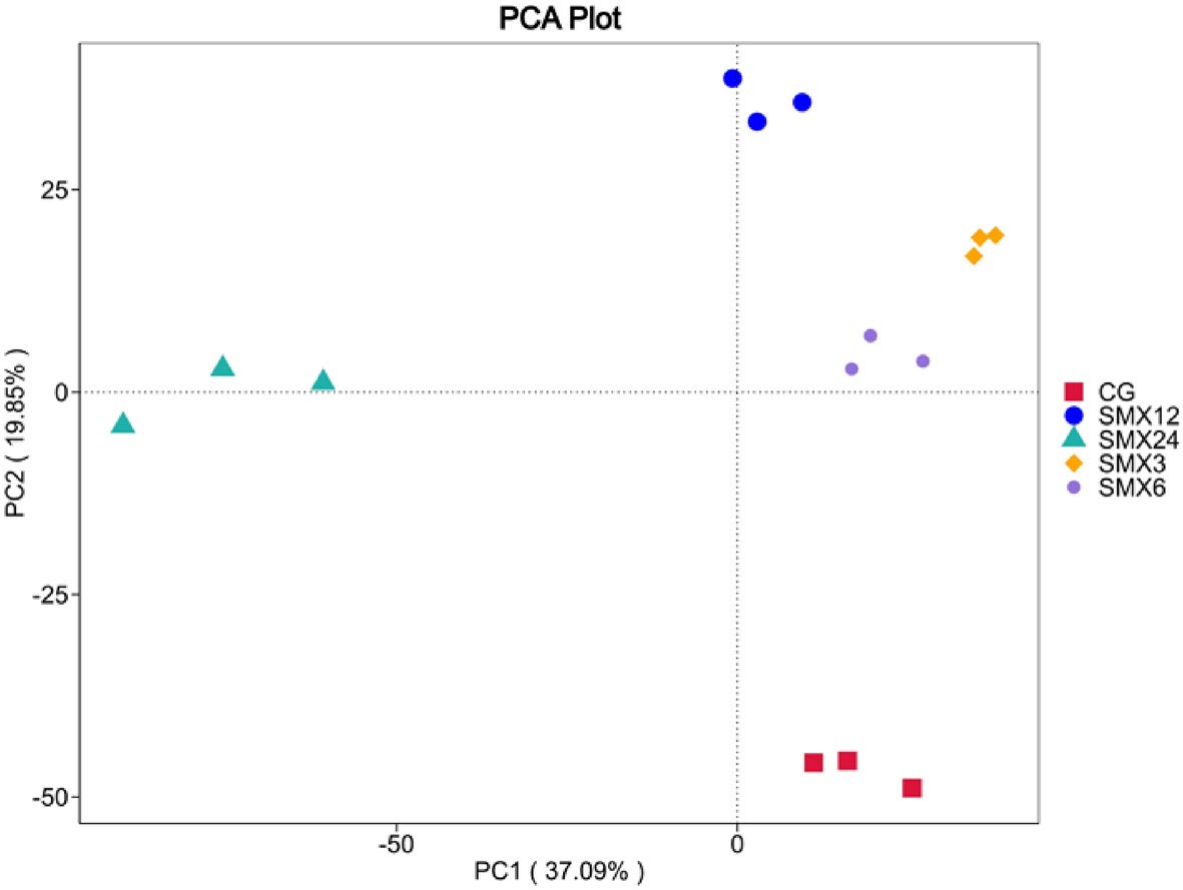
Principal component analysis of the intestinal bacterial diversity.

From Fig. 11, it can be seen that the horizontal axis represents the first principal component, and the percentage shows that the first principal component contributes 37.09% to the difference, while the vertical axis represents the second principal component, and the percentage shows that the second principal component contributes 19.85% to the difference. In the comparison of the first principal component, there were significant differences between the T24 group and other groups. In the comparison of the second principal component, there were significant differences between the T3, T6, T12, and T24 groups and the control group, indicating that SMX can significantly change the intestinal community structure, especially the distance between the T24 group and other groups, fully demonstrating significant differences from the blank group and other test groups. In contrast, the T3, T6, and T12 groups were classified into the same category, indicating differences between different groups.

#### Analysis of relative abundance of gut microbial species

**Figure 12:**
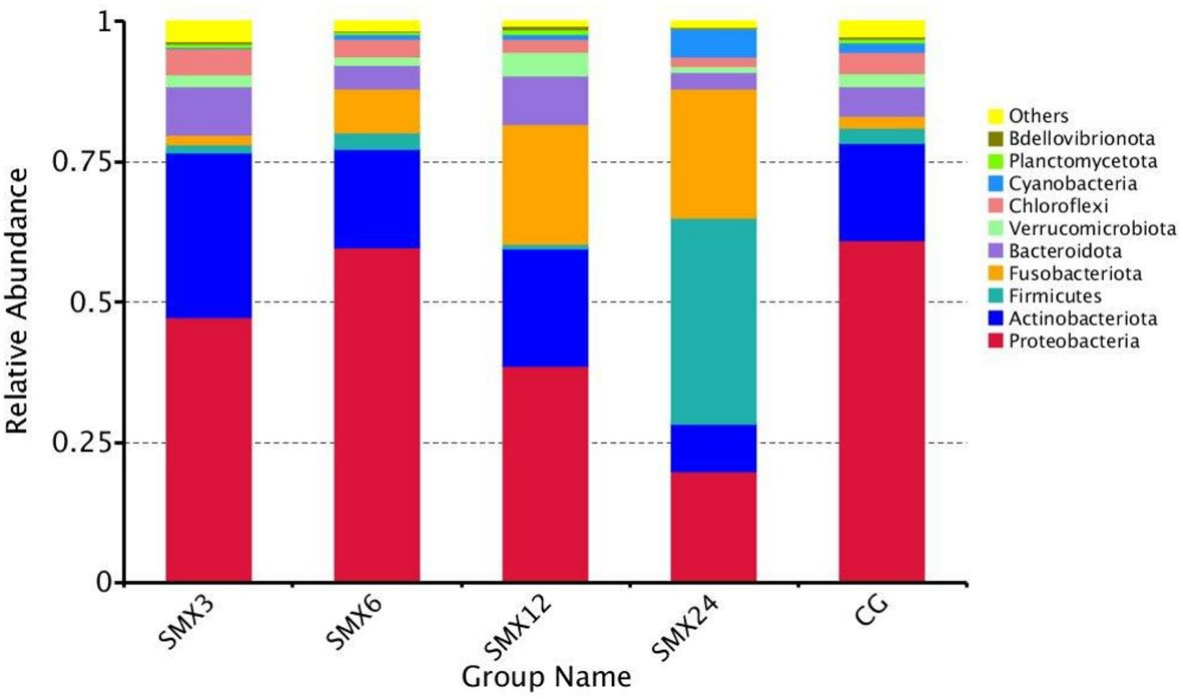
The relative abundance of each group at the phylum level.

Analyzed the gut microbiota structure exposed to SMX for 8 weeks, and selected the top 10 species based on the species annotation results of the maximum classification level abundance of each subgroup, generating a cumulative bar chart of relative species abundance, as shown in Fig. 12. At the phylum level, the main bacterial phyla include Proteobacteria, Firmicutes, and Actinobacteriota, while other bacterial phyla include Fusobacteriota, Bacteroidota, Errucomicrobiota, Chloroflexi, Cyanobacteria, Planctomycetota, and Bdellovibrionota. The results showed that the relative content of Proteobacteria in the T3, T6, T12, and T24 groups was significantly lower than that in the control group (*P* < 0.05). The relative contents of Firmicutes, Cyanobacteria, and Verrucomimicrobiota in the T3, T6, and T12 groups were lower than those in the control group. The relative contents of Actinobacteriota, Bacteroidota, and Chloroflexi in the T24 group were significantly lower than those in the control group (*P* < 0.05), and decreased with increasing concentration. The relative content of Fusobacteriota in the T6, T12, and T24 groups was significantly higher than that in the control group (*P* < 0.05).

**Figure 13:**
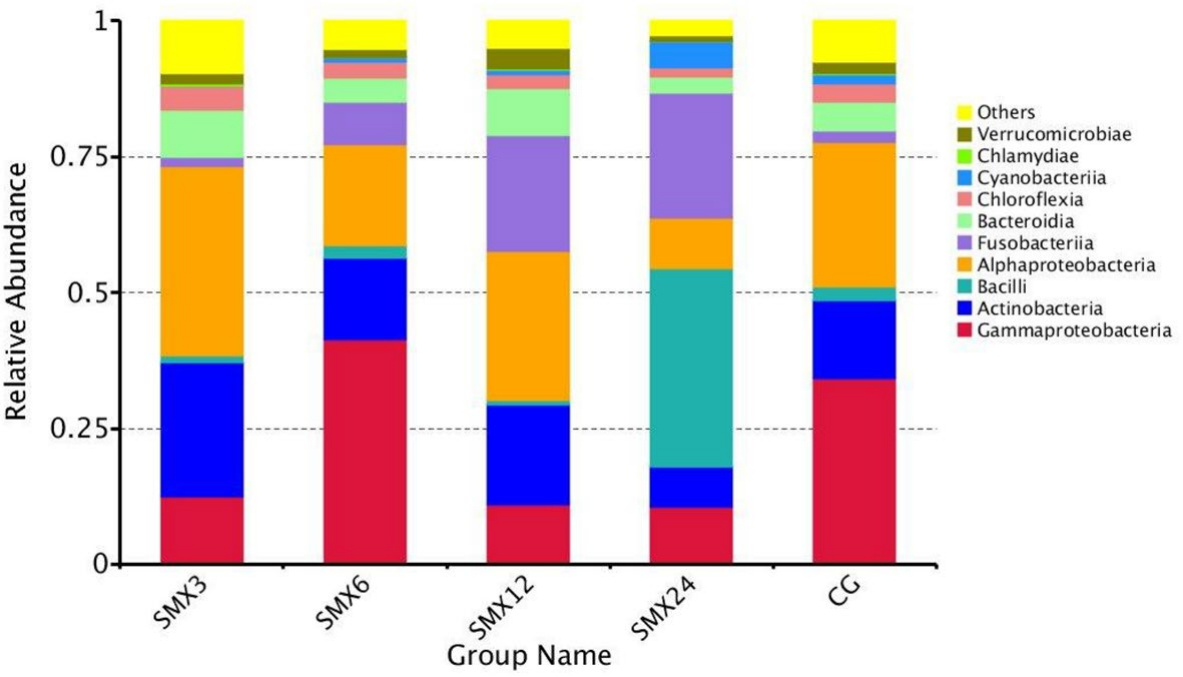
The relative abundance of each group at the class level.

As shown in Fig. 13, at the class level, γ-Proteobacteria, Actinobacteria, Bacilli, α-Proteobacteria are the four main classes of bacteria, along with Fusobacteriia, Bacteroidia, Chloroflexia, Cyanobacteriia, Chlamydiae, and Verrucomicrobiae. It was found that the relative content of γ-Proteobacteria in the T3, T12, and T24 groups was significantly reduced compared to the control group (*P* < 0.05). The relative content of Actinobacteria in the T24 group was significantly reduced compared to the control group. The relative content of Bacilli in the T3, T6, and T12 groups was significantly higher than that in the control group (*P* < 0.05). The relative content of Chloroflexia, Chlamydiae, and α-Proteobacteria in T6, T12, and T24 groups was lower than that of the control group.

**Figure 14:**
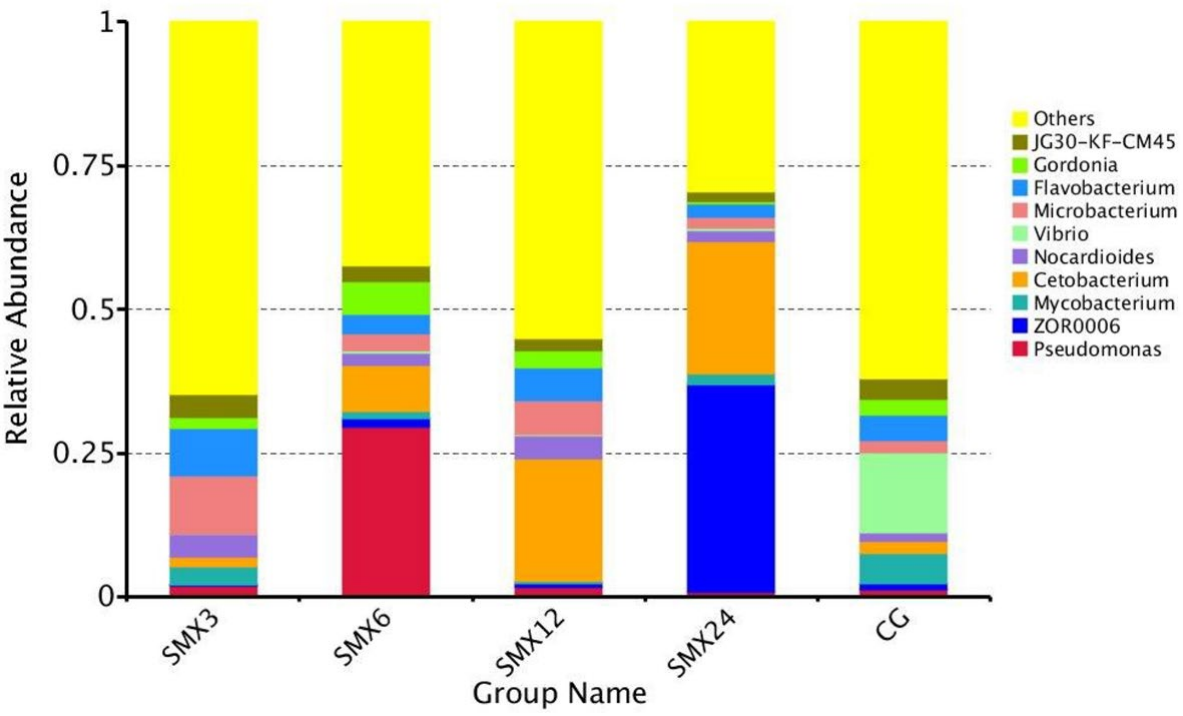
Relative abundance of each group at the genus level.

The relative abundance at the genus level was shown in Fig. 14, mainly composed of Pseudomonas, Mycobacterium, Acetobacterium, Nocardioides, Vibrio, Microbacterium, and Flavobacterium. The results showed that the relative content of Pseudomonas species in the T3, T6, and T12 groups was higher than that in the control group. The relative content of Flavobacterium in the T3, T6, and T12 groups was significantly higher than that in the control group (*P* < 0.05). The relative content of Acetobacterium in the T6, T12, and T24 groups were significantly higher than those in the control group (*P* < 0.05). The relative contents of Mycobacterium and Vibrio in all SMX concentration groups were lower than those in the control group, while the relative contents of Nocardioides and Microbacteria in all SMX concentration groups were higher than those in the control group.

#### Analysis of significant differences between groups

**Figure 15:**
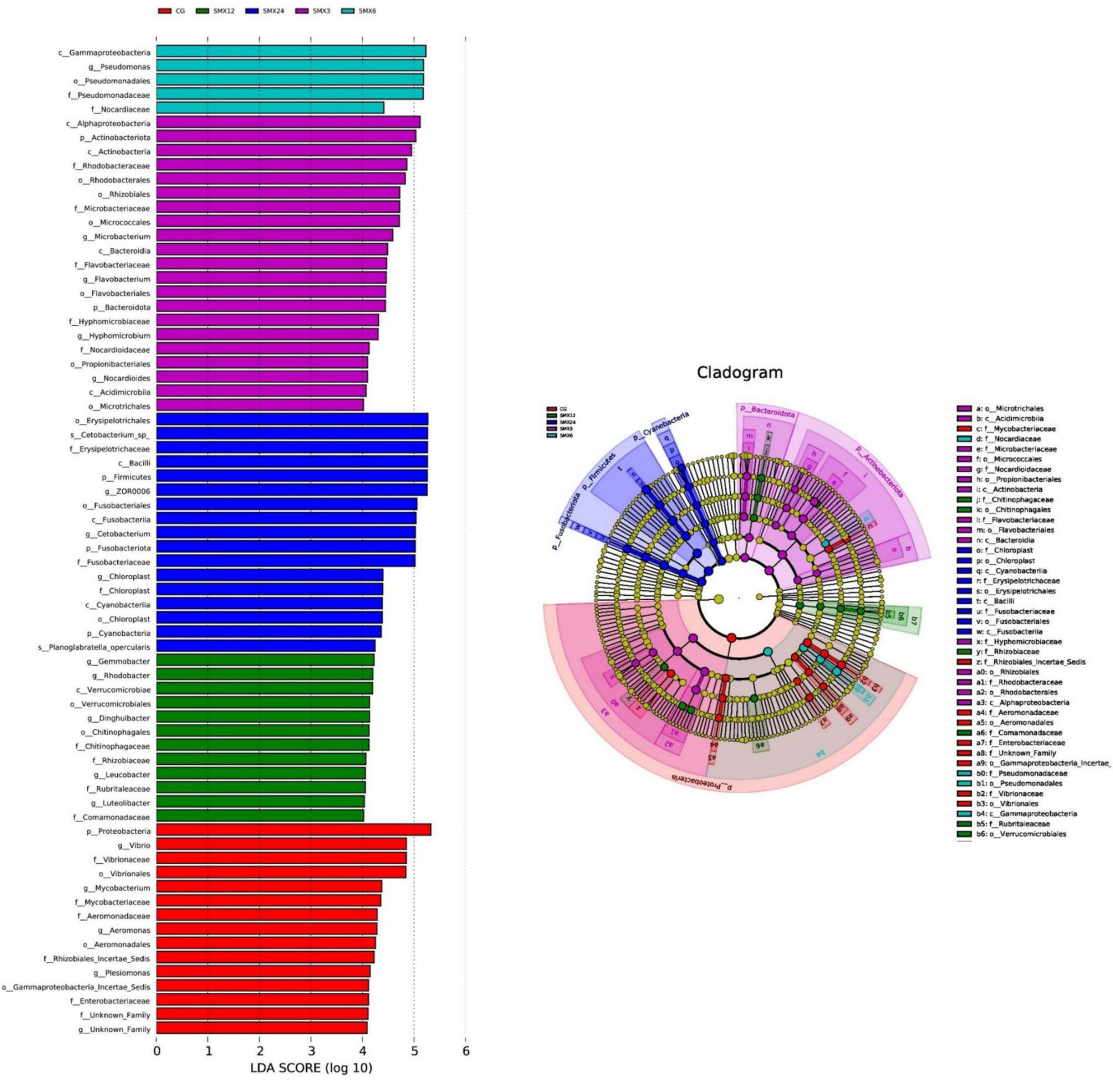
Analysis of significant differences gut microbiota between groups.

The results of significant species differences were shown in Fig. 15. The microbial communities of the control group mainly include Proteobacteria- γ-Proteobacteria—Vibrio genus of the Vibrio family, Proteobacteria—γ- Proteobacteria—Aeromonas genus of the Aeromonas family, Proteobacteria—γ-Proteobacteria—Enterobacteriaceae, and Actinobacteriota- Actinobacteria—Mycobacteriaceae—Mycobacterium; The microbial communities of the T3 group mainly include Bacteroidota- Bacteroidea—Flavobacteriaceae—Flavobacterium, Proteobacteria—α-Proteobacteria—Rhodobacterales—Rhodobacteraceae, Actinobacteriota—Actinobacteria—Propionibacteria—Nocardioidaceae—Nocardioides. The microbial communities of the T6 group mainly include Proteobacteria—γ- Proteobacteria—Pseudomonadales—Pseudomonadaceae—Pseudomonas. The microbial communities of the T12 group mainly include Bacteroidota—Bacteroidia—Manphaga crustaceans—Chitinophaga, and the genus Verrucomicrobia—Verrucomicrobiales. The microbial communities of the T24 group mainly include Firmicutes—Bacillus – Erysipelotrichales—Erysipelothrichaceae—Erysipelothrix. The species with significant differences between groups are mainly Proteobacteria, Firmicutes, Actinobacteriota and Bacteroidota.

### Residue of SMX in muscle tissue

This study showed that the residual amount in muscle tissue increased with the increase of SMX concentration. The SMX content in muscle tissue of T3 and T24 groups was 1.93 µg/kg and 3.64 µg/kg, respectively. Maximum residue detected (3.64 μg/kg fish weight) does not exceed the maximum residue of fish in animal foods (100 μg/kg fish weight).

## Discussion

### The effects of SMX on the growth of adult zebrafish

The concentration of antibiotics in aquatic environments is usually low, but long-term exposure to low concentrations of antibiotics can damage the antioxidant defense system and tissue cells of aquatic organisms. Regarding the impact of antibiotic exposure on fish growth, studies have shown that SMX can significantly inhibit the growth of zebrafish juveniles^1,14^. However, Hu et al. found that exposure to SMX (100 μg/L), there was no significant effect on the growth of Nile tilapia^15^, and Yan et al. also found that SMX of 2–20 μg/L does not alter the growth status of zebrafish^16^. Therefore, it is speculated that the toxicity sensitivity of juvenile and adult fish to SMX is different, and SMX may inhibit the growth of juvenile fish without affecting the growth of adult fish. Karci et al. selected 7 quinolone antibiotics for corresponding research on black headed fish, and the results showed that antibiotics below 10 mg/L had no significant effect on the growth of black headed fish^17^. This experiment is similar to the above research results, which showed that there was no significant difference in body weight between the exposed SMX group and the control group, and growth indicators will not change with the increase of concentration. Therefore, it can be inferred that SMX with a concentration not higher than 24 mg/L has no obvious inhibitory effect on the growth of zebrafish.

### The effect of SMX on the activity of antioxidant enzymes in zebrafish

The antioxidant system is a unique defense and protective mechanism of organisms to eliminate endogenous and exogenous toxins^18^. Organisms rely on antioxidant systems to promote reactions and alleviate oxidative damage using specific antioxidant enzymes. When stimulated by external toxic substances, the activity of antioxidant enzymes in the body is stimulated^19^. Regarding the effect of antibiotics on the antioxidant performance of fish, Tian^20^ found that SMZ has an inhibitory effect on the activity of SOD, CAT, and GSH-Px in zebrafish^20^. This study suggests that high-dose SMZ induces excessive ROS production in fish, oxidizes and modifies the active site residues of SOD, leading to a loss of SOD activity and function. In the study of Hu et al., after 30 days of exposure to 100 μg/L SMZ, the SOD activity and the levels of glutathione in the liver of tilapia significantly decreased compared to the control group, while the content of MDA significantly increased^15^. Related studies have also shown that after 7 and 30 days of SMZ exposure, the MDA content of zebrafish was significantly higher than that of the control group^1,21^. The results of this experiment indicate that the changes in the activities of SOD, CAT, and GSH-PX induced by SMX exposure exhibit a synchronous downward trend, which is particularly evident in the liver. Among them, the SMX of T12 and T24 groups can significantly inhibit SOD activity of zebrafish, which is caused by the excessive ROS produced by zebrafish under SMX stress after continuous exposure to water for 8 weeks. Organisms eliminate ROS produced by consuming their own enzyme substances, leading to a decrease in SOD activity^22^. In this experiment, the MDA contents of the liver in the T6, T12, and T24 groups was significantly increased compared to the control group, accompanied by a significant decrease in GSH-Px activity. This may be due to SMX inducing oxidative stress in zebrafish, producing excessive ROS, leading to lipid peroxidation and the formation of lipid peroxides (such as MDA)^23^, which disrupted the GSH-Px structure in tissue cells and greatly inhibited GSH-Px activity. Shen et al. also found changes in SOD activity and MDA content in the muscles of adult zebrafish exposed to 25 mg/L cefotaxime sodium for 3 and 6 days^24^, similar to the results of this study.

### The effect of SMX on the relative expression levels of antioxidant and immune related genes

This result shows that the mRNA levels of SOD, GSH-Px, and CAT genes in the liver are consistent with the overall trend of enzyme activity after antibiotic exposure, indicating that SMX may affect the activity of enzymes encoded by SOD, CAT, and GSH-PX genes by regulating transcription. In contrast, the mRNA levels of SOD, GSH-Px, and CAT genes in gill slightly deviate from the overall trend of their enzyme activity, indicating that the physiological response of the antioxidant system to oxidative stress varies in different tissues. Exposure to antibiotics in liver tissue can cause severe oxidative stress damage, and in gill tissue, antibiotic stress can also cause a certain degree of oxidative damage, mainly due to a decrease in glutathione levels^25^. Regarding the issue of inhibition of gene expression such as SOD, GSH-Px, and CAT, Kobayashi et al.^26^ believe that due to exogenous antibiotic stress, Nrf2 is induced to bind to antioxidant response element (ARE), activating the extensive expression of antioxidant genes and inducing the binding of Keap1 and Nrf2, inhibition of antioxidant related gene activation and inhibition of antioxidant gene expression^27^. It should be noted that in the T24 group of this experiment, the CAT activity in gill tissue was lower than that of the control group, but there was no significant difference in the corresponding gene expression. Meanwhile, the SOD activity in the gill of the T6 group was lower than that of the control group, and there was no significant difference in gene expression levels. This may be related to the time lag between transcription and translation, as well as post translational modifications^28,29^.

When the living organism is faced with various stressors, such as damage and invasion of pathogenic bacteria, it can induce tissue inflammatory reactions^30^. IL-1 β and IL-8 have the ability to attract and activate white blood cells, while TNF- α is the important inducer of IL-8 and IL-1 β, and plays a crucial role in activating neutrophils and macrophages. This study showed that the gene expression of IL-8, IL-1β and TNF-α in the liver and gill tissues of each SMX concentration group showed a consistent trend, with a concentration dependent upregulation. Among them, the T24 group was the most significant, indicating a great immune response in the liver. Related studies have shown that oxidative stress reduces immune defense ability, and ROS is a key signaling molecule that induces pro-inflammatory cytokines^31,32^. Therefore, it can be inferred that the gene expression of IL-8, IL-1β and TNF-α in each SMX concentration group is upregulated, indicating that SMX exposure induces oxidative stress, producing ROS and stimulating the occurrence of immune response. Meanwhile, ROS activates the TLR pathway, induces immune cell activation, and stimulates the secretion of inflammatory factors^33,34^.

### The effect of SMX on the morphology of the intestinal and liver tissues

In this experiment, as the exposure time to SMX in water increases, it causes varying degrees of damage to intestinal tissue, leading to abnormal changes in intestinal tissue. The main manifestation is oxidative damage to intestinal cells, which intensifies and leads to cell necrosis, further leading to vacuolization of muscle cells. At the same time, cell necrosis leads to damage to the integrity of the tissue barrier^35^. While, immune cells acquire substances in the cavity, induce inflammatory reactions, and induce abnormal upregulation of the number of goblet cells, Goblet cells play an important role in the host's defense against physical and chemical damage caused by the attachment and invasion of endogenous and exogenous stimuli or microorganisms^36^. The destruction of intestinal villi weakens the protective effect of intestinal mucosa, suppresses intestinal resistance, and makes fish more susceptible to external pollutants^37^, further reducing their resistance to the outside world and affecting nutrient digestion and absorption^38^. Their immune and antioxidant abilities are also weakened^39^.

Different researchers have different opinions on the causes of tissue damage caused by antibiotics. Ni et al. believes that oxidative stress and loss are important causes of tissue damage^40^, while Liu et al.^41^ believes that adverse environmental stress on oxygen free radicals produced by intestinal tissue can attack intestinal cell membranes, leading to oxidative damage. Based on the research results of this paper, it is speculated that intestinal cell damage may originate from oxidative damage caused by lipid peroxidation of intestinal cell membranes. At the same time, there are other studies that are similar to the results of this study. For example, Puerto et al. found that microcystins cause damage to mucosal epithelial cells and villous shedding in the intestinal tissue of tilapia^42^. In addition, Zheng^43^ and Zheng et al.^44^ also observed varying degrees of damage to the intestinal tissue by treating crucian carp and zebrafish with SMX, respectively. Moreover, Ni et al. also pointed out that exposure to maduramicin in zebrafish can lead to intestinal villus detachment and an increase in goblet cells^40^. The above research results indicate that exposure to SMX has an impact on the digestion and absorption capacity of zebrafish.

Most sulfonamide are prone to acetylation reactions in the liver, resulting in high concentrations and affecting the enzyme activity of liver tissue, leading to histological changes. This result indicated that as the concentration of SMX increases, the damage to the liver tissue of zebrafish becomes more severe. At concentrations of 3, 6, and 12 mg/L, the main manifestations are liver tissue congestion, liver cell vacuolization, increased intercellular matrix, and infiltration of inflammatory cells. At the concentrations of 24 mg/L, in addition to the above damage, there is also nuclear shrinkage and collapse, followed by nuclear pyknosis and nuclear lysis after staining, this may be due to SMX activating the apoptosis pathway and terminating nucleic acid transcription. Rodrigues et al. found that long-term oxidation–reduction imbalance in fish bodies can lead to oxidative damage to tissues and cells, leading to a decrease in intracellular nutrient metabolism rate and deposition of cellular substrates, resulting in vacuolar lesions^45^. Furthermore, Ozaki et al. also showed that cell death caused by severe oxidative damage can stimulate the body's immune defense and induce inflammatory cell infiltration^46^. Zheng et al. showed that after exposure to 90 µg/L SMX in zebrafish compared to the control group, some liver cells showed edema degeneration and showed a small amount of vacuolization^44^. In addition to irregular cell morphology and loose intercellular contact, the 450 µg/L SMX group also observed nuclear pyknosis, severe glassy degeneration and vacuolization of liver cell cytoplasm. In addition, the cells around the central vein undergo hydroponic degradation, with some cells exhibiting congestion or even nuclear debris.

### The effect of SMX on the gut microbiota of zebrafish

The gut microbiota is closely related to the health of the gut and host^47^. The gut microbiota is also considered an important indicator for evaluating the toxicity of environmental pollutants^14^. Many studies have shown that antibiotics can have some short-term and long-term effects on the balance of gut microbiota^48^. This result showed that there were significant changes in the gut microbiota composition in all experimental groups compared to the control group. As the content of SMX increased, the number of ASVs in zebrafish gradually decreased, and the Observed-otus, Shannon, Chao1, and Pielou-e indices significantly decreased, especially in the highest concentration of 24 mg/L. The results of intestinal sequencing showed a decrease in the chao1 index, indicating that specific beneficial microbiota in the intestine were disrupted by SMX^49^ and further altered the host's growth and immune performance^50^. The Shannon index of each group significantly decreased, indicating an uneven distribution of species and affecting the balance of the intestinal microenvironment. This is consistent with the research results of Zheng^43^, which found that the proportion of bacterial OUT in the gut samples of different carp treated with different concentrations of sulfadiazine was higher, and the diversity of gut microbiota in each sample was significantly reduced. After being treated with different concentrations of SMX in this experiment, the content of Firmicutes and Proteobacteria in the T3, T12, and T24 groups was significantly lower than the control group, while the content of Fusobacteria was significantly higher than that in the intestinal without SMX stress. At these concentrations, SMX inhibited the growth of Firmicutes and promoted the growth of Fusobacteria. Studies have shown that microorganisms in the phylum Firmicutes can promote the metabolism of gut microbiota and enhance energy uptake during the dietary process^51^. We found that exposure to SMX causes a decrease in the abundance of Firmicutes in the gut, which may lead to a decrease in the uptake of nutrients by gut microbiota, which is not conducive to the growth and development of the host. Proteobacteria is a phylum that is sensitive to dietary intake and contains some pathogenic bacteria. The sudden decrease in relative abundance of Proteobacteria in the intestine suggests an imbalanced and unstable microbial community structure, which may further promote inflammation or invasion of exogenous pathogens^52^.

The Xanthobacterium is a common bacterial genus in the intestines of fish, and is also a conditional pathogen of many fish diseases. The results of this experiment showed that compared to the control group, the abundance of species belonging to the genus Flavobacterium in the samples exposed to SMX was significantly higher. We speculate that although there is no linear correlation between the abundance of these species and antibiotic concentration, antibiotic exposure can lead to the accumulation of pathogenic Flavobacterium species in zebrafish, ultimately leading to health-related complications. This experiment speculates that sulfamethoxazole alters the composition of the gut microbiota and inhibits the growth of beneficial microorganisms at high concentrations. Cluster heat map and PCA analysis showed that the zebrafish in the T24 group showed the greatest changes in the composition of intestinal bacterial flora, with the farthest difference in spatial distribution compared to the control group.

This experiment compared the differences in gut microbiota under different concentrations of SMX in detail. In the comparison of differences between groups, pathogenic bacteria—Vibrio, Aeromonas genus, and Mycobacterium appeared in the control group, but not in the experimental group, indicating that SMX inhibited Vibrio, Aeromonas, and Mycobacterium. As the concentration increased, the abundance of common conditioned pathogen increased, such as Favobacterium and Erysipelothrix, which can cause local infections in animal bodies when immune function is low. Therefore, the results of this study fully demonstrate that SMX also has a dual polarity, inhibiting the relative abundance of certain conditioned pathogen, while also providing an upward space for the relative abundance of drug-resistant pathogen. This phenomenon has the most serious impact on the T24 group. The results of this study are similar to Zheng’s^43^ study on the effect of sulfadiazine on the intestinal flora of allogynogenetic crucian carp. This study found that the beneficial bacteria in the gut of crucian carp decreased and the abundance of potential pathoge increased. As the concentration of SMX increases, potential pathogen disorders may occur, leading to microbial metabolic disorders in zebrafish in the experimental group^53^, indicating that SMX is harmful to the microbial balance of the gut microbiota.

### Residue of SMX in muscle tissue

Antibiotic residue refers to the ability of pollutants to accumulate in organisms from the environment, and then transmit and accumulate in the food chain. After 8 weeks of exposure to SMX in this experiment, the results showed that the SMX residue content increased with the increase of SMX exposure concentration in the water body, but there was no proportional relationship between the fish body and water body concentration. Therefore, the residual situation of SMX in zebrafish was not linearly related to the exposure concentration level. This is consistent with the results of drug accumulation content in zebrafish under different exposure concentration groups of SMX after 8 days of research by Xu et al.^54^. Hou^55^ studied the bioaccumulation and excretion of sulfamethoxazole in juvenile sturgeon muscles, and the results were similar to those of this experiment^55^. As the dosage of sulfamethoxazole increased, the degree of enrichment of sulfamethoxazole in sturgeon muscles also increased, but the enrichment ability showed a downward trend.

Fish are senior consumers of aquatic ecosystems and an important source of protein for humans. This result show that under exposure to a maximum concentration of 24 mg/L SMX, the residual content of antibiotics in zebrafish muscles still does not exceed the maximum limit standards in most countries. It is calculated that when the SMX content in water exceeds 253.16 mg/L, the SMX content in muscles may exceed the national standards, and pose potential risks to human health. Although the residue does not exceed the standard, a certain concentration of SMX can have a negative impact on the antioxidant, immune function, and intestinal flora of zebrafish, thereby affecting the health of aquatic animals.

## Conclusion

The chronic toxicity test found that SMX had no obvious inhibitory effect on the growth of zebrafish when the concentration was below 24 mg/L SMX. Under different concentrations of SMX stress, oxidative damage and immune system disorder were caused to the liver and gill of zebrafish, with the 12 and 24 mg/L concentration groups being the most significant. SMX causes varying degrees of pathological changes in both intestinal and liver tissues, mainly manifested as a significant decrease in intestinal villus height, widening of villus width, significant thinning of muscle layer thickness in the 24 mg/L group, increased number of goblet cells, liver tissue congestion, inflammatory cell infiltration, vacuolization, and severe pyknosis and dissolution of liver nuclei. Moreover, with the increase of SMX concentration, tissue damage worsens. As the content of SMX increases, the abundance of common pathogen increase. SMX can significantly reduce the diversity of intestinal flora, and while inhibiting the growth of some pathogen, it also provides an increasing space for the relative abundance of drug-resistant pathogenic bacteria.

## Data Availability

All data generated during this study are included in this article.
